# Toxic Combustion Product Yields as a Function of Equivalence Ratio and Flame Retardants in Under-Ventilated Fires: Bench-Large-Scale Comparisons

**DOI:** 10.3390/polym8090330

**Published:** 2016-09-03

**Authors:** David A. Purser

**Affiliations:** Hartford Environmental Research, Hatfield, Hertfordshire AL95DY, UK; david-purser@ntlworld.com

**Keywords:** toxic products, combustion, carbon monoxide, hydrogen cyanide, flame retardant, equivalence ratio, fire, polyamide, cellulosic, polyisocyanurate

## Abstract

In large-scale compartment fires; combustion product yields vary with combustion conditions mainly in relation to the fuel:air equivalence ratio (Φ) and the effects of gas-phase flame retardants. Yields of products of inefficient combustion; including the major toxic products CO; HCN and organic irritants; increase considerably as combustion changes from well-ventilated (Φ < 1) to under-ventilated (Φ = 1–3). It is therefore essential that bench-scale toxicity tests reproduce this behaviour across the Φ range. Yield data from repeat compartment fire tests for any specific fuel show some variation on either side of a best-fit curve for CO yield as a function of Φ. In order to quantify the extent to which data from the steady state tube furnace (SSTF [1]; ISO TS19700 [2]) represents compartment fire yields; the range and average deviations of SSTF data for CO yields from the compartment fire best-fit curve were compared to those for direct compartment fire measurements for six different polymeric fuels with textile and non-textile applications and for generic post-flashover fire CO yield data. The average yields; range and standard deviations of the SSTF data around the best-fit compartment fire curves were found to be close to those for the compartment fire data. It is concluded that SSTF data are as good a predictor of compartment fire yields as are repeat compartment fire test data.

## 1. Introduction

### 1.1. Combustion Conditions, Toxic Product Yields and Equivalence Ratios in Compartment Fires and Bench-Scale Tests

In large-scale flaming compartment fires, yields of combustion products, including most toxic products, vary with the combustion conditions mainly in relation to the fuel:air equivalence ratio (Φ) (Equation (1)) and the effects of gas-phase flame-retardants, especially halogens. Yields of products of inefficient combustion, including the major toxic products CO, HCN and organic irritants, increase considerably as combustion changes from well-ventilated (Φ < 1) to under-ventilated (Φ = 1–3) [3,4,5,6,7,8].

(1) $$\Phi =\frac{actual\;fuel\;to\;air\;ratio}{stoichiometric\;fuel\;to\;air\;ratio}$$

Products of efficient combustion, notably CO_2_, NO and NO_2,_ decrease as the equivalence ratio increases, while the yields of other products, such as acid gases, remain approximately constant across the range.

Although the equivalence ratio is the major determinant of yield in flaming fires, both in compartment fires and bench-scale experiments, the global temperature environment, both in the flame zone and upper layer, also affects yields due to the effects on chemical kinetics and equilibrium states, so for given equivalence ratios in under-ventilated fires, CO yields differ in pre- and post-flashover conditions [7,8,9,10]. Similarly, there is evidence that for given equivalence ratios, the oxygen concentrations may affect yields [7]. The presence of flame retardants, in particular gas-phase halogens, can also have a marked effect on product yields by favouring inefficient combustion, even under well-ventilated combustion conditions [3,5,6,11].

The pyrolysis rates and the generation of gas-phase fuel products from materials involved in fires depend on the heat radiation to fuel surfaces, but also on physical and chemical aspects of the solid or liquid fuel composition and structure. Subsequent combustion of the airborne products then depends on the combustion conditions and is independent of the original source material.

Toxic hazards in fires depend on the concentrations of key airborne toxic species available for inhalation, which in turn depend on the fuel pyrolysis (mass loss) rate, the yields of toxic products and the dispersion of the fire effluent. While toxic gas concentrations can be measured directly in full-scale or large-scale fire experiments, for the results to be understood in terms of combustion conditions and related to different fire scenarios, it is important that the equivalence ratio is included among the parameters measured. For fire dynamics modelling, the calculated equivalence ratio in combination with appropriate expressions for product yields as a function of equivalence ratio can then be used to calculate predicted toxic gas concentrations in different fire scenarios.

It is particularly important that test measurements and hazard assessments include under-ventilated combustion conditions (Φ > 1), because the majority of fire injuries and deaths result from exposure to smoke from under-ventilated fires [3,5,12].

One way to obtain data for the relationship between Φ and product yields is to carry out a large number of repeated and appropriately-designed full-scale or large-scale compartment fires for wide ranges of different fuels. These need to include separate tests under different combustion conditions, ranging from well-ventilated fires to pre- and post-flashover under-ventilated fires, with detailed measurements of combustion product concentrations and yields as a function of Φ. While a number of such experiments have been reported for a small number of fuels, involving different compartment configurations (including ISO 9705 rooms, room-corridors and variable height hoods), the available dataset is limited by the cost and complexity of such experiments, which are unlikely to become practical on a routine basis [3,9,10,11,12,13]. Another limitation of large-scale tests is their inherent variability.

In order to obtain the necessary data from a large range of fuel materials for input to hazard models and for accurate measurement of combustion chemistry under closely-specified combustion conditions, it is therefore important that bench-scale tests are developed to reproduce the main parameters determining the yields of combustion gases and particulates from gas-phase pyrolysis products in compartment fires, including the ability to combust them across the Φ range [14]. Comparisons between the yields of combustion products in bench-scale tests and compartment fires for the same or similar fuels of different generic types can then be used to determine how well the effluent yields measured in the bench-scale test represent those in the large-scale compartment fires under comparable combustion conditions. The main requirements for any candidate bench-scale test are:

- Ability to reproduce a range of flaming set equivalence ratios over the range Φ ~ 0.5–3, which includes under-ventilated fires;
- Ability to provide yield data as a function of fuel mass loss;
- Ability to provide a hot effluent plume or upper layer over the range 300–850 °C;
- Ideally, to provide control of oxygen concentration in the air entering the combustion zone;
- Demonstrated ability to produce effluent yields comparable with the average and ranges of variability of yields obtained in compartment fires for a variety of fuels under equivalent combustion conditions across the Φ range.

### 1.2. Challenges with Measurement of Equivalence Ratios and Yields in Compartment Fires

Measurement of equivalence ratios and yields in compartment fires presents a number of uncertainties and complexities. A commonly-used configuration is of a fire compartment room with the effluent plume filling an upper layer, then flowing out from a vent, such as an open doorway, the effluent and entrained air passing into a calorimeter hood for measurement of volume flows, mass flows and composition. This arrangement was used for the “TOXFIRE” (Guidelines for Management of Fires in Chemical Warehouses) project [15,16,17]. For such experiments, one way for measuring yields is to express the mass flow of products in the hood as fraction of the mass loss rate for the fuel. The equivalence ratio in the fire compartment is then calculated as the global equivalence ratio (GER) from the mass flow of air entering the fire compartment and the fuel mass loss rate. Another method, also used for the TOXFIRE project, is to sample from the upper layer inside the room near the doorway. The sampled atmosphere was analysed for gases and a Φ meter used for direct measurement of the equivalence ratio [18]. Both methods are subject to inaccuracies due to local variations in the combustion conditions and temporal variations in fuel and air entrainment, especially during periods when the fuel mass loss rate and combustion conditions are changing [10]. Depending on the fire size and upper layer temperature, significant oxidation due to secondary combustion may occur in the doorway plume, which entrains air as it rises into the calorimeter hood, so that the yields and equivalence ratios measured outside the compartment differ from those inside the compartment. Another concept more related to the actual combustion conditions, though more difficult to measure, is the plume equivalence ratio, defined as the fuel mass-flow rate divided by the air mass-entrainment rate into the plume below the upper layer, normalized by the stoichiometric ratio for the fuel [9,10].

With respect to measuring and predicting the combustion chemistry, the most important determinant is the equivalence ratio in terms of the fuel mass loss rate and the mass of air actually mixing with the pyrolysis products in the combustion flame zone. Some further oxidation may occur beyond the flame zone if the upper layer temperature is high enough, but for non-flashed over fires, with upper layer temperatures below approximately 500 °C, oxidation is limited beyond the immediate fire plume, so that any air entrained into the cooler upper layer outside this region is essentially a diluent and does not participate significantly in the combustion chemistry. Some diluent air may also be entrained around the fire plume or more remotely, near the room doorway or beyond. Since this air is included in the estimate of the equivalence ratio, it may therefore give a false (low) estimate of the true “combustion” equivalence ratio (the equivalence ratio in the chemical combustion zone). In practice, based on reaction kinetics and measurements in combustion experiments, it is considered that in fuel-rich flames (Φ > 1), almost all oxygen is consumed [11,13,19]. On this basis, where combustion of a discrete fuel package in an under-ventilated compartment fire produces a fuel-rich upper layer, any oxygen also present is likely to have been entrained beyond the combustion zone, so that it may be treated as diluent air and excluded from the estimate of the equivalence ratio. The result of applying this correction is to slightly increase the equivalence ratio and thereby provide a small right-shift in the yield curve for CO as a function of equivalence ratio. For post-flashover under-ventilated fires, the upper layer temperatures may be high enough to facilitate continued oxidation of unburned fuel beyond the flame zone, with very low resulting oxygen concentrations in the upper layer fire plume [9,19].

### 1.3. Comparing Compartment Fire and Bench-Scale Data

As a result of the variables described and the inherent variability of conditions in compartment fires, the yield data as a function of Φ from repeat compartment fire test measurements for any specific fuel show some variation on either side of a best-fit curve. For a bench-scale measurement to be considered valid, ISO 16312-1 [20] requires overlap between the ranges of yields measured in a bench-scale test and those measured under the equivalent combustion conditions in a compartment fire. To determine the extent to which combustion gas yields measured in a bench-scale test are representative of those in compartment fires under equivalent combustion conditions, it is therefore inappropriate to consider a single measured result in a compartment fire test as a “true” value to which a bench-scale result should be compared. Rather, it is necessary to compare a set of bench-scale results with the average and distribution of yield measurements from repeated large-scale tests conducted over a range of combustion conditions and equivalence ratios.

The bench-scale steady state tube furnace (SSTF, ISO TS19700) [1,2,21] has been developed specifically to decompose test specimens under flaming combustion conditions over a range of equivalence ratios, temperatures and oxygen concentrations, enabling toxic product yields to be measured over a range of combustion conditions occurring in compartment fires.

In order to quantify the extent to which yield data from the SSTF represent those measured in compartment fires under different combustion conditions, the range and average deviations of SSTF data for CO yields from the compartment fire best-fit curves have been compared here to those for direct compartment fire measurements for six different fuels and for generic post-flashover fire CO yield data from a variety of fuels. Comprehensive datasets for intermediate to large-scale experiments on individual fuels conducted over a range of equivalence ratios are limited, but sets of usable published data have been identified for the six different fuel materials, which have been used for these SSTF comparisons. The six polymeric materials used for the compartment fire experiments were polyamide, polypropylene, wood, polymethylmethacrylate, medium-density fibreboard and polyisocyanurate foam.

For these experiments, all of the materials were in rigid form rather than as textiles, but chemically-similar analogues are used in textile fibres, including polyamide, polypropylene and polyethylene, cellulosics and acrylics. All except the cellulosics melted prior to combustion. Furthermore, since gas-phase combustion of pyrolysis products is independent of the source, the SSTF-compartment fire yield comparisons reported here for rigid products are also considered applicable to textiles with similar chemical compositions. Results for textile materials tested in the SSTF over a range of combustion conditions have been reported by Purser and Purser [7,11] and shown to be similar to those from rigid fuels, both thermoplastic and char forming.

With regard to elements with flame-retardant properties, of the materials reported in this study, two (polyamide and polyisocyanurate) had a significant nitrogen content, and polyisocyanurate also contained chlorine, while the other materials lacked elements conferring flame-retardant properties. The effects on combustion behaviour are compared for these and to published SSTF data from other flame-retarded materials [7,11].

## 2. Materials and Methods

### 2.1. Compartment Fire Experiments

Although comprehensive datasets for intermediate to large-scale experiments on individual fuels conducted over a range of equivalence ratios are limited, sets of usable published data have been identified for six different fuel materials. Details of these tests are reported in the original publications cited, with the test conditions summarised in Table 1. For each material tested in the compartment fires, tests were also carried out in the SSTF on the same polymers or chemically-similar materials with the same empirical formulas. The apparatus and experimental methods used by different authors for the compartment fires differed somewhat, but in each case, the test materials were combusted under flaming conditions over a range of measured equivalence ratios. The first two materials, polyamide 6.6 (PA66) and polypropylene (PP), were burned in floor pans in an ISO 9705 room by Blomqvist and Lonnermark [15]. The room ventilation was controlled by varying the area of an opening in the upper doorway. The next two materials, wood and polymethylmethacrylate (PMMA), reported by Gottuk and Lattimer [9], were also burned on a room floor, but controlled ventilation was provided from below via a distribution plenum. The effluent plume passed out of the room via a vent near the ceiling level. The results from these experiments are compared to those of Beyler [22] for wood (Ponderosa pine) and PMMA. For these experiments, the fires were burned in the open laboratory, and an upper layer was formed in a cylindrical hood and plenum system. The mass of air entrained into the plume, and hence, the equivalence ratio, was controlled by varying the height of the collecting hood above the fire. The final two materials, medium-density fibreboard (MDF) and polyisocyanurate foam (PIR), were tested by Purser and Purser [7,11] in two different compartment fire configurations. For one set of experiments, an ISO 9705 room was used as for the Blomqvist and Lonnermark experiments, but for these experiments, the ventilation was controlled by varying the width of the sliding door panel. The tests were performed as room corner tests. The ignition source was a crib constructed from the test material and placed in a rear corner, with the same test material used to line the walls. For these experiments, the fires were therefore not confined to a discrete fuel package on the floor, but had the potential to ignite and spread across the wall linings. A second set of experiments was performed on MDF and PIR using a half linear scale ISO 9705 room connected via a variable width doorway opening to a corridor. For these experiments the fuel packages were in the form of cribs placed on the floor in the centre of the room.

For all of the compartment fire and SSTF experiments, the purpose was to measure post-ignition flaming decomposition of the gas-phase pyrolysis products from the different fuels. The methods used for igniting the fires are not described in most cases. Normal practice for such compartment fire experiments is to use a small solid or flammable liquid ignition source, such as methenamine or methylate spirits. For Purser and Purser’s experiments, the cribs were ignited using low density fibreboard sticks soaked in 300 mL methylated spirits. For the SSTF, all specimens auto-ignited in the furnace.

For the SSTF experiments, tests were performed by Blomqvist and Lonnermark on the same PA66 and PP as used for their compartment fires. The results are also compared to SSTF data by Purser et al. [7,21] on PA66 and PP sourced separately and also on polyamide 6 (PA6) and polyethylene (PE). For their wood compartment fires, Gottuk and Lattimer used spruce, while Beyler used Ponderosa pine. Scot’s pine (*Pinus sylvestris*) was used for the SSTF experiments. Separately-sourced PMMA samples were used for the compartment fires and the SSTF experiments. For Purser and Purser’s work on MDF and PIR, the samples from the same specimens were used for the compartment fires and SSTF experiments.

Table 2 shows the composition of the materials tested in the SSTF. The carbon, hydrogen, nitrogen and chlorine contents were measured by Butterworth Laboratories using in-house elemental microanalysis methods (Butterworth Laboratories Method—BLM) BLM 0G, BLM 5 and BLM 267. The oxygen content where appropriate was calculated by difference. The measured compositions compared well to the empirical formulae for pure polymers, as shown in Table 2. For polymethylmethacrylate (PMMA), low density polyethylene (LDPE) and polyamide 6.6 (PA66), the measured compositions were within 2%–3% of empirical values, so yield calculations were based on the empirical formulae, which were considered most accurate. For wood, medium-density fibreboard (MDF) and polyisocyanurate (PIR) analysis data are shown. These values were used for SSTF yield calculations.

Polyamide 6.6 (PA66) and Polypropylene (PP): For the TOXFIRE project [15,17], individual fuels were combusted as discrete pool fire fuel packages in an ISO 9705 room [23] under different ventilation conditions obtained by varying the size of the room inlet vent. The room had one opening, 0.8 m wide 2.0 m high, centrally located at one end. The ventilation conditions inside the room were controlled by sealing the lower part of the opening with slabs of non-combustible fibreboard, which gave a reduced opening of height 0.89, 0.68, 0.56 and 0.45 m, for the development of under-ventilated conditions. The fuel was put into square pans of 1.2 m^2^ for polyamide 6.6 and 1.4 m^2^ polypropylene. Four tests were carried out, burning between 55 and 75 kg of PA 6.6 and 60 kg of PP, using a load cell to measure the mass loss. The composition of the effluent and equivalence ratio were measured in the calorimeter hood and in effluent sampled from the upper layer inside the room doorway. A Φ meter was used to determine the equivalence ratio of the fire by adding oxygen to the fire effluent sampled inside the room, passing the mixture over a catalyst at 900 °C, then measuring the resulting oxygen concentration [18]. Data for two pedigree polymers, polypropylene and polyamide 6.6, have been used for the comparison with the results for the same specimens in the SSTF by Blomqvist and Lonnermark [15] and for the same polymers (but separately sourced) by Stec et al. [21,24] Comparisons have also been made with SSTF results for polyamide 6 [7,11], which has the same empirical formula as polyamide 6.6, but a slightly different structure. Data are also shown for the comparison for low density polyethylene (LDPE), which has the same empirical formula as polypropylene [11], but a slightly different structure.

Wood and PMMA: Data for compartment fire tests on wood and PMMA are reported by Gottuk and Lattimer [9,10,25]. For these experiments, discrete fuel packages were combusted on the floor of a 1.2 × 1.2 × 1.55 m high compartment. Air was supplied at the floor level from below via a plenum and the effluent exhausted via a vent 20 cm below the ceiling. Effluent was sampled in the compartment from the upper layer below the ceiling level. The wood used was spruce. Φ was calculated as the ratio of the measured mass-loss rate of the fuel and the measured air-mass inflow, divided by the stoichiometric fuel:air ratio. Other experiments were carried out by Beyler on Ponderosa pine and PMMA [22], also using discrete fuel packages (cribs) combusted under a 1.02 m-wide hood. Air entrainment into the fire plume (and hence, the equivalence ratio) was controlled by lowering the hood to different levels. The plume equivalence ratio Φ was determined by measuring the total gas flow into the hood, the measured flow and upper layer composition. For the SSTF, experiments were carried out on separately-sourced PMMA and Scot’s pine wood (Table 2).

Medium-density fibreboard (MDF) and polyisocyanurate foam (PIR): Specimens of medium-density fibreboard and PIR were combusted as wall linings in an ISO 9705 room at the Building Research Establishment (BRE) under a range of ventilation conditions obtained by setting different door widths [11,26]. For the ISO 9705 room experiments, the walls were lined with MDF board or PIR-aluminium foil-faced panels. The standard propane burner was replaced by MDF (16 kg) or PIR cribs (2.07 kg) as the ignition sources, designed to provide a heat release rate of 300 kW. Different ventilation conditions were achieved by reducing the door width from 800 to 400, 280, 200 and 100 mm, resulting in 100%, 50%, 35%, 25% and 12.5% of full ventilation. To obtain yield data, fire parameters were measured in the upper layer inside the ISO room (0.4 m from the ceiling and 1.0 m from the door end of the room). Grab samples were taken using Tedlar bags, bubblers and on glass fibre filters. Data were also captured in the calorimeter duct. Bag samples for the estimation of fuel gases and equivalence ratios were taken as far as possible during periods of steady state combustion. Equivalence ratios were calculated using the oxygen depletion method as for the room-corridor experiments. The equivalence ratio and pyrolyzed fuel mass concentrations were calculated by the secondary combustion of effluent samples with the measurement of total oxygen required for complete combustion and the measurement of total fuel carbon as CO_2_. Compartment fire temperatures were measured with thermocouple trees inside the room.

**Figure 1:**
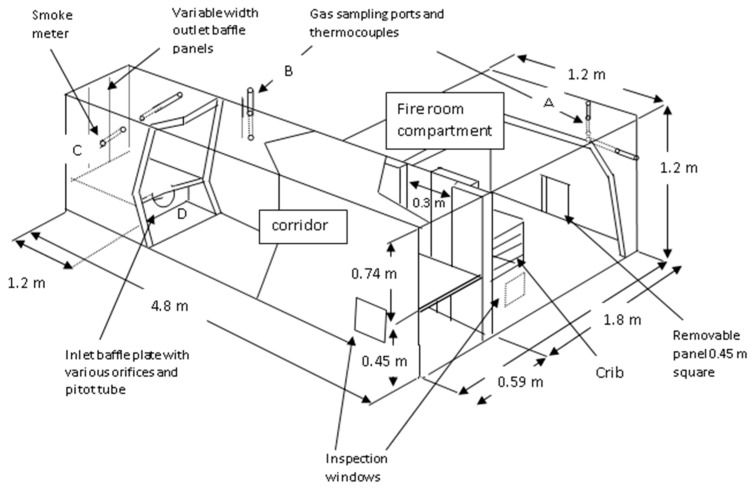
Room-corridor rig (after Purser and Purser [7]).

In other experiments, the same materials were combusted as a discrete crib in a half linear scale ISO room corridor rig [7,11,26]. The “room” had linear dimensions half those of the ISO 9705 calorimeter (Table 3; Figure 1). The corridor (4.8 m long) had a horizontal partition along its length to limit mixing of incoming air with outgoing fuel-rich fire effluent and prevent secondary combustion.

The rig was constructed from heat-resistant mineral board (“Supalux”) panels on a wooden frame. To minimise heat losses it was lined internally with ceramic blanket (room ceiling) and externally with PIR foam panels. A full height (1.2 m) adjustable sliding panel, which opened to a maximum of 30 cm in width for maximum ventilation conditions, was inserted between the room and corridor. MDF board and PIR foam cribs (mass 4–5 kg) were placed in the centre of the room on a layer of bricks to allow ventilation at the base. The MDF cribs used 120 sticks 25 × 2 × 1.22 cm in 12 layers 29.5 cm high. The PIR cribs were of 159 sticks 60 × 3.8 × 3.8 cm in 16 layers 63 cm high. Ventilation was controlled by varying the “door” panel width between 2.5 and 30 cm in 14 experiments. Effluent samples were taken from a point 12 cm below the ceiling and 20 cm from the back and side walls of the fire room and from the upper corridor (Figure 1). Samples were taken continuously for CO_2_, CO and O_2_, in the fire room and corridor, with bubbler samples for HCN and grab samples (in Tedlar bags) taken during periods of steady state combustion for the measurement of total hydrocarbons and total airborne fuel. The total airborne fuel and total oxygen demand were measured by passing a bag sample through the secondary oxidising furnace (diluting the sample with additional air if necessary). This was also used for the calculation of the equivalence ratio (Φ) under which atmosphere samples were formed according to Equation (2):

(2) $$\Phi =\frac{O_{2}required(\%)}{O_{2}supplied(\%)}$$

where: *O_2_ supplied* (%) = ambient concentration (assumed to be 20.95%) and *O_2_ required* (%) = the total oxygen depletion required for complete combustion of the fully-oxidized fuel products released in the furnace.

The yield calculations were based on measurements obtained during the steady state flaming plateau for each fire test. Specimens of the same MDI and PIR were tested in the SSTF.

### 2.2. Steady State Tube Furnace Experiments

**Figure 2:**
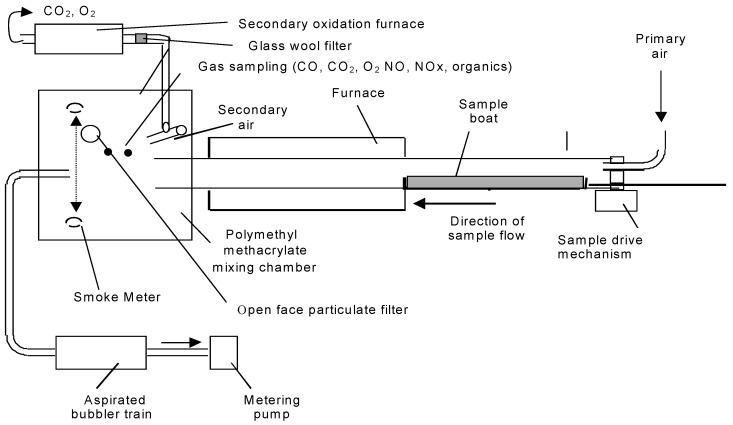
ISO TS19700 steady state tube furnace (SSTF).

The tube furnace apparatus (Figure 2) and experimental method were carried essentially as described in ISO TS19700 [2]. A specimen of material in strip form was introduced into the tube furnace at a constant rate. A current of air was passed through the furnace over the specimen to support flaming combustion. The effluent was expelled from the tube furnace into the mixing and measurement chamber, where it was diluted with secondary air, then exhausted to waste. The decomposition conditions in the furnace were set using different combinations of temperature and air flow in separate runs, to model the decomposition conditions in a range of stages and types of fires as required. The aim for each experiment was to obtain stable (steady state) flaming conditions for up to 12 min (minimum of five min) during a 20-min experimental run to enable multiple measurements to be made of the composition of the effluent in the mixing and measurement chamber. The specimen was weighed before and after the test to enable the calculation of the fuel mass charge and mass loss rate. From the data obtained, it was possible to calculate the yields of each toxic product and the actual equivalence ratio under which the combustion was carried out. This then enabled the relationships between toxic product yields and equivalence ratio to be investigated.

For these experiments, a test specimen mass of 25–50 mg/mm was used, and air-flow rates were varied between 2 and 13 L/min in order to cover the range of combustion conditions (equivalence ratios) required. For each test run, the specimen was spread evenly along the furnace boat in granular or pellet form or as short segments. Experiments were conducted under constant steady flaming conditions (verified by observation) at a furnace temperature of 650 °C. For PIR, it was necessary to increase the furnace temperature to 700 °C in order to obtain steady flaming.

The aim of each experiment was to maintain steady flaming for separate runs over a range of equivalence ratios from Φ = 0.5–2.5 at a furnace temperature of 650 °C (700 °C for PIR) to represent pre-flashover combustion conditions over the range from well-ventilated (ISO 19706 Stage 2) [14] to under-ventilated (ISO 19706 Stage 3a). Additional experiments included a higher temperature (850 °C) to represent post-flashover under-ventilated combustion conditions (ISO 19706 Stage 3b). Another variation was the use of 10% or 12% oxygen/nitrogen mixtures instead of air. By this means, it was possible to compare yields of CO and other products under the same equivalence ratios, but at different oxygen concentrations. The combustion products were diluted to a standard 50 L/min, and sampled from the mixing and measurement chamber, where other parameters were measured. The combustion products cooled as they passed through the tube beyond the furnace and down to <35 °C as they entered the mixing chamber, preventing further oxidation. Data available include fuel mass loss, heat of combustion, smoke optical density, yields of toxic species (CO, HCN, NO_*x*_ particulates, etc.) and O_2_ depletion. Data on total airborne unburned fuel content and total unburned hydrocarbons were obtained by further oxidation of the diluted fire effluent using a small secondary oxidizing tube furnace at 900 °C, with measurement of CO, CO_2_ and O_2_. CO_2_ and CO were measured dry by non-dispersive infrared analysers (NDIR) and O_2_ by a paramagnetic analyser, from the mixing chamber and secondary furnace. Mixing chamber NO and NO_2_ were measured by chemiluminescence, HCN by spectrophotometric analysis, HCl, HBr and SO_2_ by ion chromatography of bubbler solutions, smoke particulates by optical density and gravimetrically using glass fibre filters.

Experiments were set up to provide nominal equivalence ratios between 0.5 and 2.5 using appropriate fuel:air ratios calculated from the stoichiometric oxygen demand for complete combustion of the entire material or product under test. After each run, the equivalence ratio actually achieved was calculated from the total oxygen consumed for complete combustion of the airborne fuel products in the secondary furnace (basically, a Φ meter method), summed with that calculated for oxidation of the particulate sample (soot) to give the total oxygen required for the combustion of the airborne fuel during the sample period according to Equation (2). For materials undergoing complete combustion, the actual Φ values obtained were very close to the nominal values, but for materials forming a carbonaceous char residue, a proportion of the fuel was unavailable for gas phase combustion, so that the achieved Φ values calculated from oxygen depletion were somewhat lower than the nominal values, although very close to nominal values calculated assuming that residues were 100% carbon.

### 2.3. Data Comparisons

For the compartment fire tests, the data for CO yield and, where appropriate, HCN yield have been plotted as a function of equivalence ratio for each material. A least squares best-fit curve has been fitted to each compartment fire CO dataset. The deviation of each data point for CO yield from the best-fit compartment fire curve for each dataset has been measured and the range, mean and standard deviation of the deviations calculated for each case. The deviation of each SSTF data point from each compartment fire best-fit curve has also been measured, and the range, mean and standard deviation of the deviations from the compartment fire best-fit line have been calculated. For the BRE compartment fire data, the values for equivalence ratio were calculated assuming any upper layer oxygen present together with unburned fuel was a secondary diluent not involved in combustion. For other compartment fire tests, the best-fit curves were right-shifted where appropriate to align with the SSTF curves before measuring the deviations (Φ shift in Table 4). The ranges, means and standard deviations of the deviations for the compartment fire and SSTF data were then compared. The typical shape of curves for CO yield as a function of equivalence ratio over the Φ range is sigmoid, so that CO yields are generally low below stoichiometry, with a rapid increase around Φ = 1–1.5, which then tends to level off. For curves of this general form, the best fit has been obtained using a Weibull function of the form:

(3) $$Y_{R}=k1-(1-e^{-{\left(\Phi k2/\beta \right)}^{\alpha }})/k3$$

where: $Y_{R}$ is the yield of product R (g/g), in this case CO, Φ is the equivalence ratio and the values for α, β and constants *k*_1–3_ vary between the individual materials.

For polyamide, polyethylene and PMMA, the compartment fire data did not extend to high enough Φ levels for the curve to flatten off, so that a good fit was obtained with an exponential function.

(4) $$Y_{R}=\alpha e^{-\Phi \beta }$$

Sigmoid Weibull functions were used for wood and MDF.

For PIR, a linear expression fitted the ISO 9705 room Φ data plus the lower Φ points of the room-corridor data, so that:

(5) $$Y_{R}=\alpha \Phi +\beta$$

Above Φ ~ 1.3, the behaviour of PIR was more complex, as described. The expressions fitted for each material and constants are listed in Table 4.

## 3. Results

### 3.1. Results from Compartment Fire and SSTF Experiments on Six Materials

**Figure 3:**
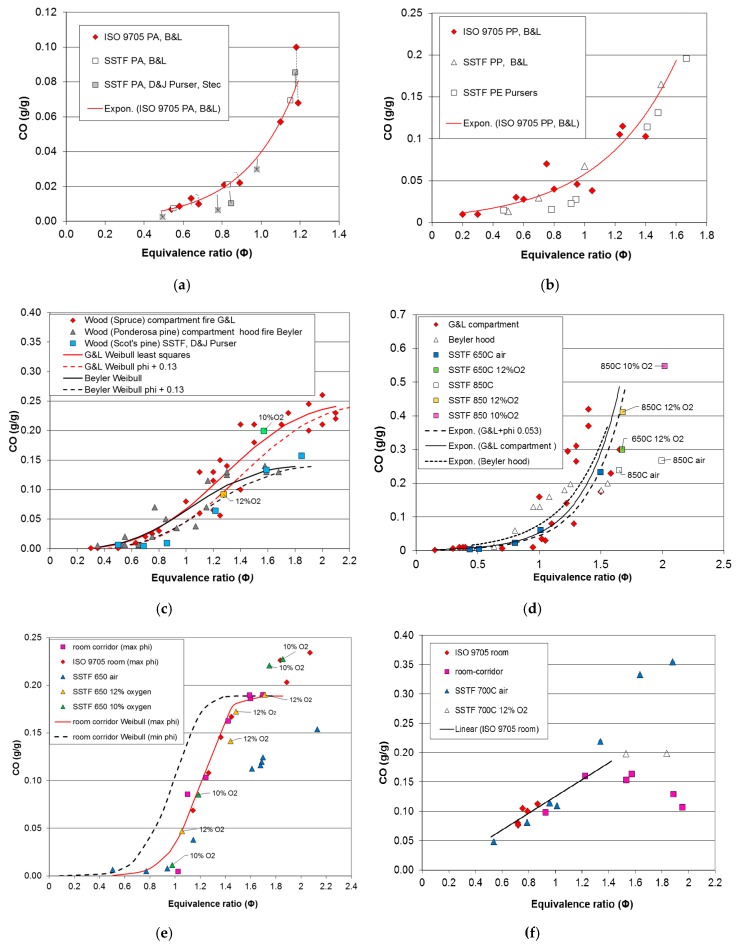
Data points and fitted curves for CO yield (g/g) as a function of equivalence ratio (Φ) for six materials measured in compartment fire experiments compared to data points for the same or similar fuels measured in SSTF experiments. (**a**) polyamide 6.6 and 6; (**b**) polypropylene and polyethylene; (**c**) wood; (**d**) polymethylmethacrylate (PMMA); (**e**) medium-density fibreboard (MDF); **(f**) polyisocyanurate (PIR). B&L, Blomqvist and Lonnermark; Expon, Exponential.

Data points and fitted curves for CO yield as function of equivalence ratio (Φ) for each of the six materials are shown in Figure 3a–f. For the compartment fires, Φ values were calculated as described in Section 2.1 and Section 2.3. The effects on comparisons for each material of secondary air entrainment are described below.

Figure 3a compares CO yields as a function of the equivalence ratio obtained by Blomqvist and Lonnermark (B&L) [15] from full-scale compartment pool fires in the ISO 9705 room under different doorway ventilation conditions for polyamide 6.6 with those obtained by the same authors using the steady state tube furnace (ISO TS19700) [2] and by Stec et al. [27]. Three points (marked “x”) are also shown for PA6 [7], which has the same empirical formula as PA6.6, but a slightly different structure. As illustrated, the method of comparison was to measure the deviations of the CO yield for each point from the compartment fire least squares fit curve. Figure 3a shows a good general agreement between the compartment fire and SSTF results over the somewhat limited Φ range measured in the compartment fire tests, to which an exponential function has been fitted (CO (g/g) = 0.001 × exp(3.721Φ)). From SSTF data obtained at higher equivalence ratios and those obtained from other SSTF and compartment fires, the CO and HCN yields curve continues to increase up Φ = 1.5, then flattens off at higher equivalence ratios, giving a sigmoid shape to the full curves with a maximum CO yield of around 0.2 g/g [27].

Figure 3b shows the comparison for polypropylene pool fires in the same ISO 9705 room measured by Blomqvist and Lonnermark [15] compared to their SSTF data for the same materials and, for comparison, those measured by D and J Purser [11] for the chemically-similar polymer polyethylene (LDPE). As for polyamide, an exponential least squares curve has been fitted to the data over the somewhat limited compartment fire Φ range. There is good agreement between the compartment fire data and both sets of SSTF data. As with polyamide, the curves for polypropylene and polyethylene reach a maximum of around 0.2 g/g CO at Φ = 1.6, then level off or decrease at higher equivalence ratios [27]. For both of these pool fires in the ISO 9705 room, there was little increase in the CO yield above well-ventilated baseline levels until just below the stoichiometric equivalence ratio of one. There is also a good direct fit with the SSTF data, which is not improved by introducing a right shift into the compartment fire best-fit curve. It therefore seems that in these compartment fire experiments, using a small, high level vent in the doorway, either there was no appreciable secondary air entrainment into the upper layer beyond the combustion zone, or the high upper layer temperatures (750–996 °C) were sufficient to facilitate oxidation reactions beyond the immediate combustion zone. The result is that the Φ values measured provide a good representation of the combustion Φ.

Figure 3c shows comparisons for wood. The compartment fire dataset shown with red diamonds is from Gottuk and Lattimer’s (G&L) chamber experiments for Spruce cribs [9,25]. This shows the classical sigmoid curve (using a fitted least-squares Weibull curve) up to a maximum of approximately 0.25 g/g CO at a measured Φ of two. Also shown is the dataset from Beyler’s hood experiments using Ponderosa pine and a fitted curve [10,22] (grey diamonds and black curve). This follows a similar pattern to Gottuk and Lattimer’s curve up to a Φ of one, but then levels off at a somewhat lower CO yield around 0.15 g/g. For both datasets, the CO yield starts to rise at measured equivalence ratios around Φ ~ 0.7, which are somewhat below stoichiometry. For the SSTF tests on Scot’s pine, the main dataset was obtained by combustion in air. The distribution fits both fire curves up to a Φ of one, but above this follows Beyler’s curve with a maximum yield of 0.16 g/g CO for Φ = 1.85. A difference from the compartment fire data is that in the SSTF, the increase in CO yield above baseline does not occur until close to stoichiometry. It is therefore considered that both Beyler and Gottuk and Lattimer’s curves may include some secondary dilution air. The Φ values for both compartment fire datasets are considered by the authors to represent the plume equivalence ratios under steady state burning conditions, but since both include secondary dilution air and excess fuel, they do not fully represent the equivalence ratio in the combustion zone, requiring a small right shift to compensate, for comparison with the SSTF data. The best fit with the SSTF data is obtained by right shifting the compartment fire curves by adding a Φ correction of +0.13, giving the dashed curves shown in Figure 3c. Minimum values for the deviations of the SSTF data then occur using this corrected fire curve. In addition to the SSTF runs in air, two tests were performed in 10% and 12% oxygen using nitrogen/air mixtures. These combustion conditions were found to give higher CO yields than for runs in air at the same Φ levels (above one) for a variety of fuels. For wood, two such test results are shown in Figure 3c, and in particular, the test run at Φ = 1.6 gave a higher CO yield of 0.2 g/g, which is more consistent with Gottuk and Lattimer’s data. The statistical comparison for Gottuk and Lattimer’s data with the SSTF data has therefore been made using the results for air below Φ = 1 and the air/nitrogen mixtures for Φ > 1. This set of SSTF data also shows agreement with data from a full-scale room burn of wood cribs by Alarifi et al. [28].

Figure 3d shows data and exponential curves for polymethylmethacrylate (PMMA). Gottuk and Lattimer’s compartment fire data [9,10] show a considerable scatter for Φ > 1, with one set of points indicating a rapid increase in CO yield up to 0.45 g/g for Φ in the 1–1.4 range and another set showing a more gradual increase to 0.3 g/g at Φ = 1.65. For Beyler’s hood results [10,22] (dotted curve), the increase in CO yields started at Φ = 0.8, increasing to a maximum of 0.2 at Φ = 1.6, and these are therefore somewhat left-shifted and lower than G&L’s yields. The SSTF results at 650 °C for air up to Φ = 1.5 lie in the middle of Gottuk and Lattimer’s dataset. One reason for the variability in Gottuk and Lattimer’s data and the difference from Beyler’s may be the higher and more varied upper layer temperatures reported by Gottuk and Lattimer’s (527–897 °C) and lower oxygen concentrations in their enclosed rig with restricted ventilation below the fire and a small high level exhaust vent. When PMMA was combusted at 850 °C in the SSTF in 10% and 12% oxygen using air:nitrogen mixtures, higher CO yields of up to 0.55 g/g were obtained, as shown in Figure 3d. The statistical yield comparisons have been made for the standard 650 °C in the air SSTF dataset using a right shift correction to the Gottuk and Lattimer’s curve of Φ = 0.053 (shown as the dashed curve in Figure 3d). As with the data from the wood experiments, this compensates for the presence of dilution air in the compartment fires not involved in the combustion process.

The final two fuels were the medium-density fibreboard and aluminium foil-faced polyisocyanurate foam. These were burned as cribs in a room-corner configuration in the ISO 9705 room, with the same materials also present as wall linings. They were then burned as cribs in the half scale room corridor apparatus [7,11,26]. For all of these experiments, different doorway openings were used to vary the fuel:air ratios between well-ventilated and under-ventilated pre-flashover combustion conditions. The equivalence ratios were calculated from the composition and total oxygen demand of effluent samples taken from the upper layer just below the ceiling level inside the doorway in the ISO 9705 room and in the corner farthest from the door in the room compartment of the room-corridor rig. For these samples, two versions of the equivalence ratios were calculated, a minimum Φ taking into account all fuel and oxygen in the sample and a maximum “combustion” Φ assuming that oxygen in the presence of unburned fuel was from secondary dilution air not involved in combustion. The points plotted for MDF in Figure 3e and the solid fitted curve are all for maximum Φ in the room-corridor experiments, showing an initial increase in CO yield around Φ = 1. The dashed line represents the minimum Φ curve, which is left-shifted by a Φ value of −0.22. During these fires, the upper layer temperature below the ceiling was 500–600 °C, being somewhat lower for the more under-ventilated fires. The CO yield datasets from both compartment fires are very similar, except that the maximum levels in the ISO 9705 room were somewhat higher, with a maximum of 0.23 g/g compared to 0.19 for the room-corridor. This may be because the ISO room tests included MDF on the walls in the hottest and most vitiated upper layer conditions, while the room-corridor tests used only cribs on the floor. Another possibility, as proposed by Pitts, is that additional CO may be formed nearer to a room doorway (where the ISO 9705 room samples were taken) as some air is entrained into a hot, fuel-rich, upper layer [29].

MDF was burned in the SSTF at a furnace temperature of 650 °C in air and in 12% and 10% oxygen using air-nitrogen mixtures. Figure 3e compares the data from the compartment fires and SSTF. The solid blue triangles give the data for combustion in air, showing a good agreement with the maximum Φ data for the compartment fires up to a Φ of 1.2, but then levelling off with a maximum value of 0.15 g/g at Φ = 2.1. When 12% and 10% oxygen mixtures were used, the yields were closer to the room-corridor and ISO 9705 compartment fire curves with a maximum level of 0.23 g/g. The presence of unburned fuel with oxygen in the compartment fire tests indicates the presence of dilution air, so that the maximum Φ provides a better estimate of the true Φ in the combustion zone. This was therefore used for statistical comparison between the room-corridor CO yields, the ISO 9705 room yields and the SSTF, using data for air/nitrogen mixtures for Φ > 1.

The behaviour of the aluminium-faced polyisocyanurate foam (PIR) in the compartment fires showed some variability (Figure 3f). In the lined ISO 9705 room, the PIR crib burned well, but involvement of the aluminium-foil faced PIR on the walls was minimal, so that the resultant fuel-air ratios and equivalence ratios for these tests were all quite low. In the half scale room corridor rig, the PIR cribs also burned well for equivalence ratios up to Φ = 1.2, with an approximately linear increase in CO and HCN yields up to this level. At higher equivalence ratios above 1.5 in the room-corridor rig, a change in combustion behaviour occurred involving a large increase in smoke and particulate yields and a decrease in CO yields, especially at higher Φ above 1.8. This change in combustion behaviour was associated with a reduction of the upper layer temperature from 550 down to 400 °C in different experiments as the equivalence ratio increased. In the SSTF, a furnace temperature of 700 °C was found necessary to obtain stable flaming, and under these conditions in air, there was a good agreement with the compartment fire CO and HCN yields up to around Φ = 1.3. At higher equivalence ratios, the SSTF CO yields in air were somewhat higher than those obtained in the room-corridor rig, although closer when 12% oxygen was used. Statistical comparisons have been made between the ISO 9705 results with the first two room-corridor data points up to Φ = 1.2, using an approximate maximum Φ to compensate for secondary air entrainment. These have been compared to the SSTF data for air.

### 3.2. Results for Uncertainty and Accuracy Analysis off Compartment Fire and SSTF Data

**Figure 4:**
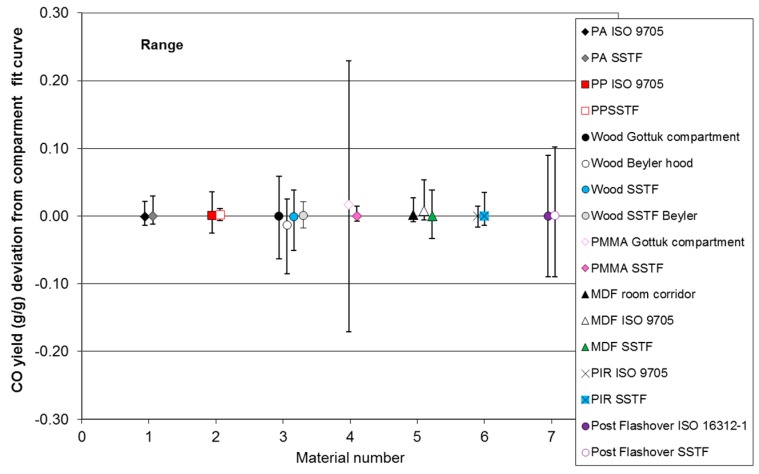
Means and ranges of deviations from the trend lines for compartment fire CO yields and from compartment fire trend lines for SSTF CO yields.

**Figure 5:**
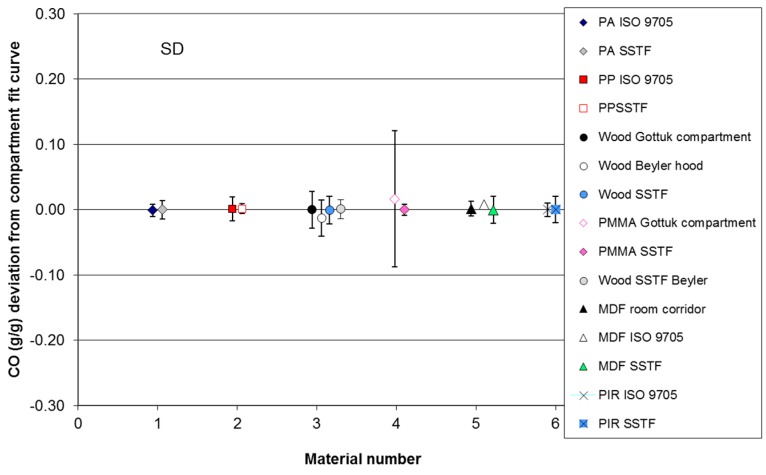
Means and standard deviations of deviations from trend lines for compartment fire CO yields and from compartment fire trend lines for SSTF CO yields.

The results of the uncertainty comparison calculations for carbon monoxide yields for each of the six materials for which compartment fire trend lines have been fitted are shown in Table 5 and Figure 4 and Figure 5. The upper part of the table shows the compartment fire data, while the lower part shows the SSTF data. For each material dataset, the average yield has been calculated, the mean and standard deviations for the deviations of the data points from each trend line and the lower and upper deviation ranges. For the compartment fire data, the deviations have been measured directly from the compartment fire trend lines. For the SSTF data, the deviations have also been measured from the compartment fire trend lines (e.g., ”Polyamide v ISO 9705” indicates comparison between the SSTF polyamide data and the ISO 9705 polyamide best fit curve), right-shifted if appropriate to compensate for secondary air entrainment as specified for each material in Section 3.1. The second column in Table 5 shows the average CO yield (g/g). This simply reflects the average values for the data points measured over the Φ range. Since the yields vary from close to zero at Φ = 0.5 up to around 0.2 g/g at Φ = 2.0, the average yield is typically around 0.1 g/g, except for polyamide and polypropylene, for which no measurements were made at the higher Φ levels.

The third column shows the mean deviation of these points from each calculated curve. This is expressed as a percentage of the average yield in the fourth column. The fifth column shows the standard deviation for the deviations of the points from the curve. The sixth and seventh columns show the range of the deviations in terms of the lowest point below the curve and the highest point above the curve. Data from ISO 16312-1 [20] for post-flashover compartment fires have also been entered into the table, showing an average CO yield of 0.24 ± 0.09 g/g (0.15–0.33 g/g). The lower part of the table shows the data for the six same or similar materials from the SSTF also compared to the compartment fire trend lines. The analysis therefore shows the extent of variation of the compartment fire data around the trend lines and the extent of variation of the SSTF data around the same compartment fire trend lines. This indicates the extent of agreement between the compartment fire and SSTF results and the suitability of the SSTF results as predictors of compartment fire values.

The results for deviation averages, ranges and standard deviations are illustrated in Figure 4 and Figure 5. Figure 4 plots the average deviation of the compartment fire values and the deviation ranges for each material (left range for each material) compared to the average deviation and range of the equivalent SSTF data (right range for each material). These are the data shown in the sixth and seventh columns of Table 5. Figure 5 shows the standard deviations.

The results for the six materials show similar mean deviations and ranges of variation and overlap between the compartment fire and SSTF results for each material, indicating that the SSTF results are as good a predictor of compartment fire yield as the repeat compartment fires themselves. For Material 6 (PMMA), the variation in the compartment fire data was greater than that for the SSTF. For the compartment fires, it is to be predicted that the mean deviation of the data from each best-fit curve should be small, provided the curve is a good fit to the data. This is indeed the case for each material, with mean deviations close to zero. Since the average deviations of the SSTF data from the adjusted compartment fire curves are also very close to zero, this demonstrates that these are also a good fit to the compartment fire data. For the six materials, the overall deviation of the SSTF data was very small at 0.0003 g/g CO, and for no material was the difference between the compartment fire and SSTF deviations from the compartment fire curves statistically significant. The fourth column of Table 5 shows that the average deviations of SSTF datasets for each material from the mean compartment fire yields are typically within a few percent of the mean yields. The ranges of the deviations and standard deviations provide a measure of the scatter of the data around the best-fit curves. As shown in Table 5 and Figure 4 and Figure 5, those for the compartment fires and the SSTF were very similar, indicating a similar variability in results for both the compartment fire and SSTF data. Overall, the deviation ranges and standard deviations of the SSTF data are lower than those of the actual compartment fire data, as shown in Table 5. This is due to the inherent variability of the conditions in compartment fires with respect to both the time during a test and the location within a compartment, while the conditions in the SSTF are maintained constant in the furnace tube during the measurement period and are more precisely defined.

For Materials 1 and 2 (polyamide and polypropylene), the comparison is somewhat limited by the upper Φ levels of the compartment fire experiments (Φ = 2 and 1.5). For Material 3 (pine wood), four ranges are shown in Figure 4 (also see Figure 3c). The first is for spruce wood burned in compartment fires by Gottuk and Lattimer [9,25]. The second is for Beyler’s hood experiments (Ponderosa pine) [10,22], giving a similar, but slightly lower range, especially at high Φ values. The third and fourth ranges are the SSTF data (Scot’s pine). The third range uses the SSTF data obtained under depleted oxygen combustion conditions at higher Φ levels compared to Gottuk and Lattimer’s under-ventilated compartment fire data and showing a close agreement. The fourth range is a comparison of the SSTF data obtained in air with Beyler’s hood data, also showing close agreement between the compartment fire and SSTF datasets. The difference between the two compartment fire datasets is considered to be related to differences in oxygen concentration and temperatures in the combustion zones.

For Material 4 (PMMA), there is significant variability in the compartment fire data, which is reflected in the wide deviation ranges and higher standard deviations, while the SSTF data are more consistent. As with the wood data, the variability in the compartment fire data is considered likely to be due to variations in oxygen concentration and temperature at given Φ levels during different experiments or at different times within experiments, while the combustion conditions within the SSTF are maintained constant.

For Material 5 (medium-density fibreboard (MDF)), the first range in Figure 4 is for the set of half scale room corridor crib fires, the second for ISO 9705 wall lining tests and the third for SSTF data (see Figure 3e). The deviation ranges for all three are small and overlap, but the room-corridor and SSTF CO yields tend to be slightly lower than the lined room results. This may be because in the lined room, some of the fuel is in the hot upper layer near the ceiling, where it may be exposed to non-flaming thermal decomposition conditions favouring CO production, while in the room-corridor rig all of the fuel is on the floor in crib form and flaming.

Material 6 is the polyisocyanurate insulation foam (PIR). For this material, comparing the data is more complicated. Essentially, PIR was found to burn in a consistent manner in the ISO 9705 room tests and also in the room-corridor tests up to a Φ of around 1.3, with an approximately linear increase in CO yield with Φ over this range. The comparisons in Table 5 and Figure 4 and Figure 5 have been made over this range, showing good agreement. At higher Φ levels above 1.5, the combustion behaviour becomes more complex, as described (see Figure 3f), so that under the low oxygen concentrations and lower temperature in the room-corridor rig and in the SSTF under reduced oxygen, the CO yield becomes lowered in favour of greater particulate formation.

The seventh comparison in Figure 4 shows differences between the average and range values for post-flashover compartment fires from ISO 16312-1 [20] compared to the average value and range for ten materials tested in the SSTF at 850 °C and Φ values in the 1.5–2 range as a measure of predicted post-flashover compartment fire CO yields (Table 6) [11]. This demonstrates the ability of the SSTF to provide a good prediction of post-flashover compartment fire CO yields. For the post-flashover yields, the data are not corrected for the carbon content of the fuels. When the data are normalised for carbon content, the CO yield ranges are reduced.

The expressions for the best-fit curves, together with the means and standard deviations for the SSTF data, provide a potential tool for engineers to predict compartment fire yields and variability for these six materials. They also illustrate the accuracy and levels of uncertainty of the SSTF data for each material as a predictor of average compartment fire values and ranges. The results show that the averages and ranges for the SSTF data are in good agreement with the compartment fire data across the ranges of equivalence ratios shown in Figure 3. There were no statistically-significant differences between compartment fire and SSTF datasets for any of the six materials.

Although in several cases SSTF data exist for higher equivalence ratios than for the compartment fire data, it is not possible to provide direct validation against the compartment fire data at these higher equivalence ratios until further compartment fire data become available. However, as shown in Table 5 and Table 6 and Figure 4, there is a good agreement between the SSTF and compartment fire data for combustion conditions in post-flashover compartment fires, which are fuel-rich and typically at high equivalence ratios.

### 3.3. HCN and Other Products of Inefficient Combustion

**Figure 6:**
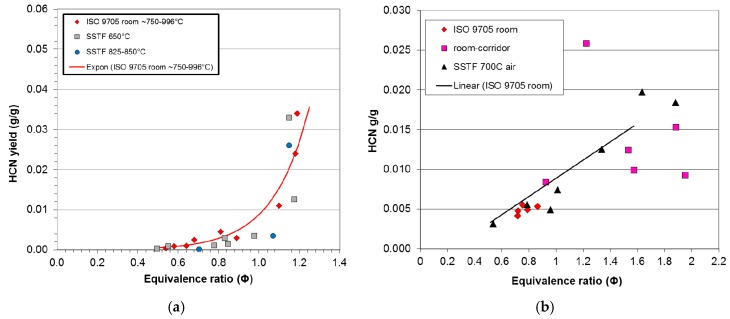
Data points for HCN yield (g/g) as a function of equivalence ratio (Φ) for two nitrogen-containing materials measured in compartment fire experiments compared to data points for the same or similar fuels measured in SSTF experiments. (**a**) Polyamide 6.6 and 6; (**b**) Polyisocyanurate.

The statistical comparison between toxic product yields in compartment fires as a function of equivalence ratio and those from the SSTF has been limited to carbon monoxide, since this is a major product of inefficient combustion for which data are available from the compartment fires and SSTF for all six materials. However, in the SSTF and a number of large-scale compartment fires, a similar sigmoid relationship between yield and equivalence ratio also occurs for other products of inefficient combustion. For all materials, these consist of a range of organic vapours and particulates, (which includes irritants, such as acrolein and formaldehyde, carcinogens, such as polycyclic aromatic hydrocarbons, and environmental contaminants, such as dioxins and furans) [11,30]. For materials containing nitrogen, there is a similar relationship between equivalence ratio and HCN yield. This is illustrated in Figure 6a,b, for which the HCN yield curves are very similar to the CO yield curves shown in Figure 3b,f. In practice, it has been found that the efficiency of conversion of fuel nitrogen to HCN is similar to that of fuel carbon to CO, so that the normalised yields of the two gases are similar [13,19]. Where halogens are present, these cause inefficient combustion over the entire Φ range. The main consequence of this is that the yields of inefficient combustion products, including CO and HCN at low equivalence ratios (Φ < 1) are increased approximately in proportion to the percentage of chlorine and/or bromine in the fuel [6]. Of the materials reported in this study, only PIR had a significant chlorine content (3.56%), which resulted in the minimum CO yield at Φ = 0.7 of 0.07 g/g compared to 0.001–0.01 g/g for the other (non-halogenated) materials.

## 4. Discussion

### 4.1. Comparison of CO Yields as a Function of Φ between Compartment Fires and the SSTF

Given the complex combustion environment in compartment fires, it is perhaps surprising that good predictions of yields can be made, not only for the major products of combustion, such as CO_2_, water and oxygen consumption, but also more “minor” combustion products, including CO and HCN (which are the most important toxic products for human fire hazards). Studies such as those of Pitts, Beyler, Gottuk, Lattimer and Alarifi [9,10,22,25,28], together with others described here [7,11,13,15,17,26,27], have demonstrated that the yields of products of inefficient combustion, especially CO, in flaming fires depend mainly on the fuel:air equivalence ratio, which is a powerful tool for the prediction of yields in compartment fires involving different fuels. These studies have also shown that the equivalence ratio is not the sole determinant of CO yields, so that upper layer temperature (pre- or post-flashover) and the local air entrainment and oxygen concentration in both flames and hot, fuel-rich, plumes also have some influence. The result is that CO yields (and those of other toxic products) tend to be somewhat higher in the hot, fuel-rich, upper layers of post-flashover fires than in the cooler upper layers of pre-flashover under-ventilated fires. CO yields are also enhanced somewhat in situations when flames are burning mainly in low oxygen environments or when some air is secondarily entrained into hot, fuel-rich, but oxygen-depleted upper layers. Another feature of under-ventilated compartment fires is that upper layer temperatures tend to be lower under the most fuel-rich conditions. This may be responsible for the change in combustion behaviour of polyisocyanurate, from mainly gaseous combustion products to enhanced particulate formation.

The primary purpose of this paper is to examine the possibility of going one step further in obtaining yield data for input to fire engineering calculations for hazard assessments; namely, the extent to which the ISO TS19700 bench-scale method (SSTF) can reliably measure CO yields (and those of other products) as a function of Φ for the combustion of fuels with different compositions, with an accuracy comparable to that possible in large-scale compartment fires. The results presented here for six fuels with different compositions, including cellulosics, thermoplastics and materials containing nitrogen and chlorine, have demonstrated a remarkable agreement between SSTF and actual compartment fire yield curves. The means, standard deviations and ranges of the deviations from the compartment fire best-fit curves were very similar for both the compartment fire and SSTF data. In addition to this, the SSTF results showed similar variants in yield with higher temperatures and different oxygen concentrations as were found in the compartment fire studies. The average SSTF CO yield data from a set of materials for under-ventilated post-flashover combustion conditions also shows a close agreement with the average and range of those reported for large-scale fire studies [11,20].

The results obtained under different tightly-specified flaming combustion conditions in the SSTF therefore not only provide a tool for replicating different compartment fire combustion conditions, but also enable the effects of varying different parameters to be measured. For the materials reported here and others published previously [3,7,8,11,21,30], it has been found that, while Φ is the main determinant of the yields of products of inefficient combustion (especially CO and HCN) in the SSTF, the furnace temperature downstream of the flame zone influences yields, with somewhat increased yields at higher temperatures for under-ventilated conditions (Φ > 1). Another factor found to influence yields for unventilated conditions is the oxygen concentration in the flame zone, which resulted in almost doubling of yields for some materials. Under these conditions, blue flames were observed, indicative of low flame temperatures favouring CO production. The findings of similar variations in yields at the same Φ levels in the wood, PMMA and MDF compartment fires indicate that similar effects are occurring in different compartment fires relating to variations in oxygen concentration in the flame zones and/or the oxidation of fuel gases in hot regions of upper layers where secondary air is entrained [9,10,11,22,25,26,28,29,31]. For lined rooms, another source of CO may be non-flaming oxidation of fuel near the ceiling.

The advantages of the SSTF over compartment fire experiments are the ability to make many accurate repeat measurements of product yields at low cost over a wide range of defined combustion conditions. However, there is still a need for a larger set of well-characterised full-scale compartment fire experiments for different fuels, both to obtain fundamental data and to provide a larger reference database against which large and bench-scale test methods can be validated.

### 4.2. Effects of Flame Retardants on the Relationship between Φ and Yields of CO and HCN

Although the effects of flame-retardancy are not the main focus of this paper, the results presented here do illustrate some useful aspects, especially when compared to SSTF results from a wider range of polymers [6,7,11]. Flame retardants affect the reaction-to-fire properties of materials (the ease of ignition and, in some cases, the heat release rate), but also the yields of toxic gases released under different combustion conditions. All of the experiments reported here used ignition sources and conditions sufficient to obtain flaming combustion, so they do not address ignitability, and the main purpose was the measurement of yields. For the nitrogen and halogen-containing polyisocyanurate (PIR), one effect of the flame retardancy was that a higher furnace temperature was required in the SSTF to obtain stable flaming than for the other polymers, while in the lined room, there was little involvement of the lined area, compared to that for MDF. Most relevant to this study was the effect on product yields. There was significant char formation with under-ventilated combustion conditions in the SSTF, possibly related to the char-forming influence of the fuel nitrogen. Furthermore, under well-ventilated combustion conditions in both the SSTF and compartment fires, the yields of both CO and HCN were higher than for other materials. This is considered to be due to the influence of gas phase chlorine compounds. When the yields of products of inefficient combustion (organics, particulates, CO and HCN) were compared for different polymers burned under well-ventilated combustion conditions (Φ < 1), they were found to be approximately proportional to the chorine and/or bromine content of the polymer [6,8,30]. These effects on char formation and CO yields were not observed with the other nitrogen-containing polymer PA66, which has a very different chemical structure and contained no halogens.

### 4.3. Implications of Results for Toxic Hazards in Fires

The development of toxic hazards in fires depends on the mass loss rate of the burning fuel and the extent of conversion of the airborne pyrolysis products to toxic species. The results of these compartment fire and SSTF experiments demonstrate that during the early, well-ventilated stages of fires, the yields of toxic products (including CO, HCN, smoke and irritants) are generally very low unless the burning fuel has a significant halogen content [3,6]. Anyway, these products rise in the buoyant plume and accumulate under the ceiling, so that during the first few minutes of most fires in occupied enclosures, the toxic hazards are generally low. However, because such fires typically occur in enclosed spaces with limited ventilation (exterior doors and windows closed), the upper effluent layer rapidly fills down towards the floor. As it does so, the air entrainment into the flame decreases, and the upper flames of the now larger fire begins to burn in the oxygen-depleted upper layer. The result is an increase of the equivalence ratio, providing under-ventilated combustion conditions (Φ ~ 1.5–2) and a large increase in the yields and concentrations of toxic species, especially CO and HCN (if the nitrogen content of the fuel is >~1% by mass) [3,6]. The result is rapid incapacitation followed by death for any room occupants or occupants beyond the room of origin exposed to the effluent, within a minute or so of exposure to these conditions. This occurs before the fire is large enough to result in flashover, but if the ventilation is sufficient to support flashover, then even larger areas of a building become untenable. For these reasons, it is vital that the correct data for toxic product yields as a function of Φ are available for input to fire dynamics calculations and computer simulations so that fire hazards are not underestimated.

## 5. Conclusions

The main conclusion of this study is that the steady state tube furnace (SSTF) provides a simple and versatile method for measuring the yields of CO and other toxic products over a wide range of defined combustion conditions, with an accuracy at least as good as repeat full-scale tests, for predicting yields in compartment fires under equivalent combustion conditions.

The results from both the SSTF and compartment fire studies demonstrate that the yields of products of inefficient combustion, and especially the major toxic gases CO and HCN, increase considerably with the equivalence ratio as the initial, well-ventilated, combustion conditions occurring in compartment fires change to under-ventilated conditions, resulting in high concentrations, leading to rapid incapacitation and death for exposed occupants. For Φ > 1, these higher yields are further increased at higher temperatures and lower oxygen concentrations, while for Φ < 1, they are increased by the presence of halogenated flame retardants.

The flame-retarded PIR foam produced higher yields of both CO and HCN for well-ventilated combustion conditions than the other polymers, but significant char formation and higher smoke particulate yields occurred for under-ventilated combustion conditions.

The validation of the SSTF and other bench-scale methods as predictors of toxic product yields for different combustion conditions and for understanding of the effects of different parameters on combustion processes would be considerably improved if the set of compartment fire experiments designed to measure effluent products as a function of equivalence ratio was increased to include more common materials, products and items occurring in buildings and transport systems.

## Figures and Tables

**Table 1:** Compartment fire apparatus and test methods used for different materials.

Material	Author	Compartment Fire Test Method
PA66 and PP ^a^	Blomqvist and Lonnermark [15]	ISO 9705 room with variable opening in the upper part of the doorway 0.8 × 0.89, 0.68, 0.5, or 0.45 m to the exterior calorimeter hood. Fuel in 1.2 or 1.4 m^2^ floor pans
PMMA and wood ^b^	Gottuk and Lattimer [9]	Chamber 1.57 m high × 1.22 m × 1.52 m. Air from below via 30.5-cm diameter duct and distribution plenum, variable area exhaust vent 20 cm below the ceiling. Fuel package in pan on the floor above the plenum
	Beyler [22]	1.0 m diameter × 0.4 m depth insulated cylindrical hood, enclosed top and sides, set at variable heights above an open burning fuel package. Effluent vented from upper 15 cm of hood into the collection plenum and duct system
MDF and PIR ^c^	Purser and Purser [7,11]	ISO 9705 room with a doorway 2 m high and width opening varied from 0.8 to 0.1 m opening to the exterior calorimeter hood. Fuel consisting of crib in floor corner configuration as the ignition source and the same fuel as the wall linings
		Half linear scale ISO 9705 room with a doorway 1.2 m high and width varied from 0.3 to 0.05 m opening to a 4.8-m corridor. Fuel as the crib in tray on the floor in the room centre

^a^ PA66 (polyamide 6.6), PP (polypropylene); ^b^ PMMA (polymethylmethacrylate); ^c^ MDF (medium density fibreboard), PIR (polyisocyanurate foam).

**Table 2:** Composition of test materials by elemental analysis and calculation from the empirical formula of the pure polymer. LDPE, low density polyethylene.

Material	Method	Elemental Composition (%)					^a^ Stoich. O_2_ Demand (g/g)
		C	H	O	N	Cl	
PA66	Analysis	62.25	9.88	16.01 ^b^	11.86		
	Empirical formula	63.68	9.80	14.19	12.38		2.33
LDPE ^c^	Analysis	85.50	14.51				
	Empirical formula	85.63	14.37				3.42
Wood (P. syl) ^d^	Analysis	49.6	6.1	44.22	0.14		1.38
PMMA	Analysis	60.33	8.14	31.53 ^a^			
	Empirical formula	59.98	8.05	31.96			1.92
MDF	Analysis	47.90	6.13	41.66	3.69	0.62	1.35
PIR	Analysis ^e^	63.5	4.98	21.8 ^a^	6.15	3.56	1.87

^a^ Stoichiometric; ^b^ % calculated by difference; ^c^ LDPE (low density polyethylene); ^d^ *Pinus sylvestris*; ^e^ includes 2.8% glass.

**Table 3:** Room-corridor rig dimensions.

Component		Length (m)	Width (m)	Height (m)
Room compartment		1.8	1.2	1.2
Corridor		4.8	0.6	1.2
Corridor section	upper section, effluent exit	–	0.59	0.74
	lower section, air inlet	–	0.59	0.45

**Table 4:** CO (g/g) compartment fire yield expressions for all materials.

Material	Rig	Expression	α	β	*k*_1_	*k*_2_	*k*_3_	Φ Shift
PA66	9705	exponential	0.000962	3.7210				0
PP	9705	exponential	0.007729	2.0133				0
Wood	room	Weibull	3.2	18	−0.001	12.6	4	0.13
Wood	hood	Weibull	3.2	14.65	−0.001	12.6	7	0.13
PMMA	room	exponential	0.00174	3.4150				0.053
PMMA	hood	exponential	0.00450	2.8379				0.053
MDF	rm-corr ^a^	Weibull	7	39	0	31	5.3	Φ_max_
PIR	9705	linear	0.1423	−0.0169				Φ_max_

^a^ rm-corr = room-corridor rig

**Table 5:** Accuracy and uncertainty of compartment fire and SSTF data in relation to best-fit curves for CO yield as a function of Φ.

Material		Average Yield (g/g)	Mean Deviation (g/g)	Mean Deviation (%)	Deviation Standard Deviation (g/g)	Range below Average (g/g)	Range above Average (g/g)
**Compartment Fire Data in Relation to Best-Fit Compartment Fire CO Curves as a Function of Φ**							
1	Polyamide ISO 9705	0.0344	−0.0011	−3.1	0.0092	−0.0126	0.0224
2	Polypropylene ISO 9705	0.0541	0.0012	2.2	0.0180	−0.0265	0.0350
3	Wood G&L ^a^ room	0.1257	0.0000	0.0	0.0280	−0.0628	0.0589
	Wood Beyler hood	0.1125	−0.0132	−11.7	0.0276	−0.0724	0.0387
4	PMMA G&L room	0.1341	0.0167	13.4	0.1043	−0.1872	0.2125
5	MDF room-corridor	0.1317	−0.0016	1.2	0.0115	−0.0102	0.0252
	MDF ISO 9705	0.1531	0.0082	5.3	0.0246	−0.0140	0.0454
6	PIR ISO 9705	0.1049	0.0000	0.0	0.0105	−0.0164	0.0148
Average		0.1063	0.0017	0.79	0.0292	−0.0503	0.0566
7	Post Flashover 16312-1	0.24				−0.09	0.09
	**SSTF Data in Relation to Best-Fit Compartment Fire CO Curves as a Function of Φ**						
1	Polyamide v ^b^ ISO 9705	0.0342	−0.0001	−0.4	0.0137	−0.0121	0.0296
2	Polypropylene v ISO 9705	0.0556	0.0016	2.9	0.0078	−0.0077	0.0094
3	Wood v G&L room	0.1250	−0.0006	−0.5	0.0214	−0.0504	0.0395
	Wood v Beyler hood	0.1134	0.0009	0.2	0.0084	−0.0073	0.0145
4	PMMA v G&L room	0.1343	0.0002	0.8	0.0146	−0.0181	0.0120
5	MDF v room-corridor	0.1314	−0.0003	−0.2	0.0208	−0.0330	0.0389
6	PIR v ISO 9705	0.1052	−0.0003	0.2	0.0200	−0.0137	0.0349
Average		0.0999	0.0003	0.42	0.0153	−0.0203	0.0267
7	Post Flashover 16312-1	0.241				−0.091	0.101 ^c^

^a^ G&L, Gottuk and Lattimer; ^b^ v, versus; ^c^ 0.2 includes glass reinforced plastic (GRP).

**Table 6:** CO yields in the SSTF simulating a post-flashover combustion furnace temperature of 850 °C Φ = 1.5–2.0

Material	CO Yield (g/g)
Polyamide (PA6)	0.26
LDPE	0.14
MDF	0.17
MDF-fire retarded	0.23
PIR	0.23
PMMA	0.34
FPU	0.28
PAN	0.15
GRP	0.44
PVC plasticised	0.17
Average	0.241
